# Oncolytic virus and CAR-T cell therapy in solid tumors

**DOI:** 10.3389/fimmu.2024.1455163

**Published:** 2024-10-30

**Authors:** Eleonora Ponterio, Tobias Longin Haas, Ruggero De Maria

**Affiliations:** ^1^ Department of Oncology and Molecular Medicine, Istituto Superiore di Sanità, Rome, Italy; ^2^ Dipartimento di Medicina e Chirurgia Traslazionali, Sezione di Patologia Generale, Università Cattolica del Sacro Cuore, Rome, Italy; ^3^ IIGM - Italian Institute for Genomic Medicine, Candiolo, TO, Italy; ^4^ Fondazione Policlinico Universitario “A. Gemelli” - I.R.C.C.S., Rome, Italy

**Keywords:** CAR T cells, oncolytic viruses, cancer, immunotherapy, solid tumor

## Abstract

Adoptive immunotherapy with T cells, genetically modified to express a tumor-reactive chimeric antigen receptor (CAR), is an innovative and rapidly developing life-saving treatment for cancer patients without other therapeutic opportunities. CAR-T cell therapy has proven effective only in hematological malignancies. However, although by now only a few clinical trials had promising outcomes, we predict that CAR-T therapy will eventually become an established treatment for several solid tumors. Oncolytic viruses (OVs) can selectively replicate in and kill cancer cells without harming healthy cells. They can stimulate an immune response against the tumor, because OVs potentially stimulate adaptive immunity and innate components of the host immune system. Using CAR-T cells along with oncolytic viruses may enhance the efficacy of CAR-T cell therapy in destroying solid tumors by increasing the tumor penetrance of T cells and reducing the immune suppression by the tumor microenvironment. This review describes recent advances in the design of oncolytic viruses and CAR-T cells while providing an overview of the potential combination of oncolytic virotherapy with CAR-T cells for solid cancers. In this review, we will focus on the host-virus interaction in the tumor microenvironment to reverse local immunosuppression and to develop CAR-T cell effector function.

## 1 From conventional therapies to CAR-T cell immunotherapy with OVs

Cancer treatment has undergone a radical transformation thanks to groundbreaking advancements in research and drug development. While chemotherapy once stood as the primary treatment, targeting rapidly dividing cancer cells, it also affected proliferating healthy cells, such as those of the epithelial and hematopoietic compartments. However, the advent of molecular profiling and understanding of cancer cell mutations has revolutionized treatment approaches. Random screening has been replaced by targeted therapies that specifically target molecular abnormalities in cancer cells (1).

Immunotherapy has rapidly ascended as a cutting-edge and highly promising domain within oncological treatments. While the response to immunotherapies varies among patients, those who do respond can achieve substantial improvements. A leading innovation in this field is Chimeric antigen receptor (CAR) T-cell therapy (2). CAR-T cell therapy involves the clinical use of adoptive cell therapies to combat cancer (3), introducing new possibilities alongside conventional treatments like surgery, radiotherapy, and chemotherapy. In this approach, a patient’s immune system is supported with infused T cells that have been genetically modified to specifically target tumor cells. This approach holds great promise and has demonstrated clinical efficacy in hematologic malignancies (4). These T cells have been modified to express a CAR. The CAR is an artificial molecule engineered to induce cytolytic T cell reactions in tumors. The CAR combines the extracellular single chain variable fragment (scFv) portion with the ability to recognize tumor-associated epitopes and the intracellular signalling domains that are required for T cell activation (3). At present, the clinical efficacy of CAR-T cell therapy is mainly limited to hematologic malignancies. Some successes were reported in preclinical studies on solid tumors but most of the CAR-T cell therapies targeting these types of cancers are yet not ready for FDA approval.

In this review we describe some of the drawbacks of CAR-T cells in solid tumors and how a combination of this therapy with oncolytic viruses (OVs) could potentially function in a synergistic manner, offering complementary and additive benefits.

## 2 CAR-T cells show limited efficacy in solid tumors, underscoring the need for new solutions

The therapy based on CAR-T cells faces challenges in targeting solid tumors, with issues related to identification of the optimal antigen, effective trafficking, infiltration, and persistence within the immunosuppressive tumor (5–8).

These issues often lead to reduced efficacy and potential toxicity (9). To address these limitations, researchers are developing advanced CAR designs that incorporate multiple costimulatory molecules, ligands, and soluble cytokines (10). Additionally, combining CAR-T cell therapy with checkpoint blockade and targeting inhibitory factors in the tumor microenvironment has shown promise in reducing T-cell exhaustion (11). Ongoing research aims to enhance CAR-T cell proliferation and tumor destruction capabilities in solid tumors by addressing mechanisms of CAR-T cell dysfunction (11). Strategies to improve CAR-T cell efficacy include enhancing tumor infiltration, persistence, and overall function (12).

### 2.1 Trafficking

After infusion, CAR-T cells need to accumulate at the tumor site. This process is favoured by interventions targeting the tumor vasculature and by promoting the expression of chemokine receptors that promote T cell infiltration (13). For instance, CAR-T cells engineered to co-express CXCR2/CCR2b (receptors for CXCL1) have shown enhanced targeting towards mesothelioma tumor cells expressing CXCL1. Similar results were obtained with αvβ3- or αvβ6-targeting CAR-T cells, which express integrins found on tumor vascular endothelium (14–18). Blocking endothelin B receptor has also demonstrated enhanced T cell infiltration into tumor lesions (19).

### 2.2 Infiltration

CAR-T cell therapy has been effective for hematological malignancies but struggles with solid tumors due to issues with infiltration and expansion (8). To overcome these challenges, researchers are exploring several strategies. One approach involves engineering CAR-T cells to express heparanase, an enzyme that breaks down extracellular matrix components, improving tumor infiltration (15). Combining CAR-T cells with vascular disrupting agents like combretastatin A-4 phosphate (CA4P) has also shown improved infiltration and efficacy in preclinical models (20). Additionally, Tian et al. (21) engineered CAR-T cells to express CXCL9, enhancing their cytokine secretion, cytotoxicity, and ability to recruit T cells while inhibiting tumor angiogenesis. Engineered CAR-T cells to express IL-8 receptors for improved migration and persistence in the tumor microenvironment (22). These innovative strategies aim to enhance CAR-T cell therapy’s efficacy in solid tumors by improving infiltration, antitumor activity, and survival outcomes in preclinical studies.

### 2.3 Tumor microenvironmental immune suppression

Tumors employ various mechanisms, including the recruitment of tumor-associated macrophages, myeloid-derived suppressor cells, and activated regulatory T cells, to create an immune-tolerant microenvironment (23). Understanding the tumor microenvironment (TME) in solid tumors reveals a complex interplay of cells and signals sustaining tumor growth and immune evasion. Strategies to counteract the immunosuppressive TME include immune checkpoint inhibitors and genetically engineered CAR-T cells to secrete immune-modulating compounds. Incorporating cytokines like IL-12, IL-18, or IL-36γ into CAR-T cells enhances bystander T cell activation, and co-stimulatory ligands such as CD40L boost nearby T cell effector functions. (24–28). Moreover, CAR-T cells expressing dendritic cell growth factors like Flt3L activate host T cells and promote an effective immune response (28). Addressing antigenic heterogeneity, researchers have developed EGFRvIII CAR-T BiTEs (29). While these approaches show promise, further research is needed to optimize CAR-T therapy in solid tumors, considering specific CAR constructs and patients’ clinical histories. Combining CAR-T therapy with checkpoint blockade holds potential (30), but requires careful consideration of efficacy and potential toxicity (31).

However, positive preclinical studies fostered new efforts that mesoCAR-T cells expressing anti-PD-1 antibodies are being evaluated in a phase II trial for treating mesothelin-positive advanced solid tumors (32).

Cell death within tumors is complex, and true “immunogenic” cell death, capable of triggering an anti-tumor immune response, is rare (33–35). Dying normal cells tend to be tolerogenic, and dying tumor cells often activate suppressive pathways, such as IDO, TGFβ, and Treg activation, hindering the immune response (36–38). Activated Tregs play a crucial role in establishing tolerance to dying tumor cells and can inhibit CAR-T cell activation through various mechanisms (39). The immunosuppressive properties of activated Tregs can limit CAR-T cell therapy effectiveness. Strategies to modulate Treg function or reduce their presence within the tumor microenvironment may enhance CAR-T cell efficacy. Combining therapies targeting both Tregs and tumor cells simultaneously could improve CAR-T cell therapy outcomes, particularly in the context of immune tolerance. Additionally, the enzyme IDO, which inhibits T-cell function, can be expressed in tumors and hinder CAR-T therapy, but targeting IDO with certain drugs may enhance the success of CAR-T treatments (40). Indeed, ongoing research, bolstered by technological advancements, offers the promise of maximizing the efficacy of immunotherapy. This entails also synergistically merging CAR-T therapy with oncolytic viruses (OVs) to address solid tumors. This evolving landscape holds significant promise for the future of cancer treatment.

### 2.4 Oncolytic viruses

OVs are a promising class of viruses that show the potential to selectively target and destroy cancer cells, leaving healthy cells largely unharmed. (41). Once inside the host cells, OVs replicate and may cause lysis of the cancer cells, or produce their primary therapeutic benefit by delivering immunostimulatory transgenes that trigger or augment an immune response against the tumor. OVs exert various effects that contribute to their anti-cancer activity, including: (i) the release of tumor antigens, (ii) the release of PAMP-like molecules, which trigger the innate immune system to recognize and eliminate infected cells, including cancer cells, (iii) the stimulation of the innate immune responses, (iv) the release of proinflammatory cytokines.

**Figure 1:**
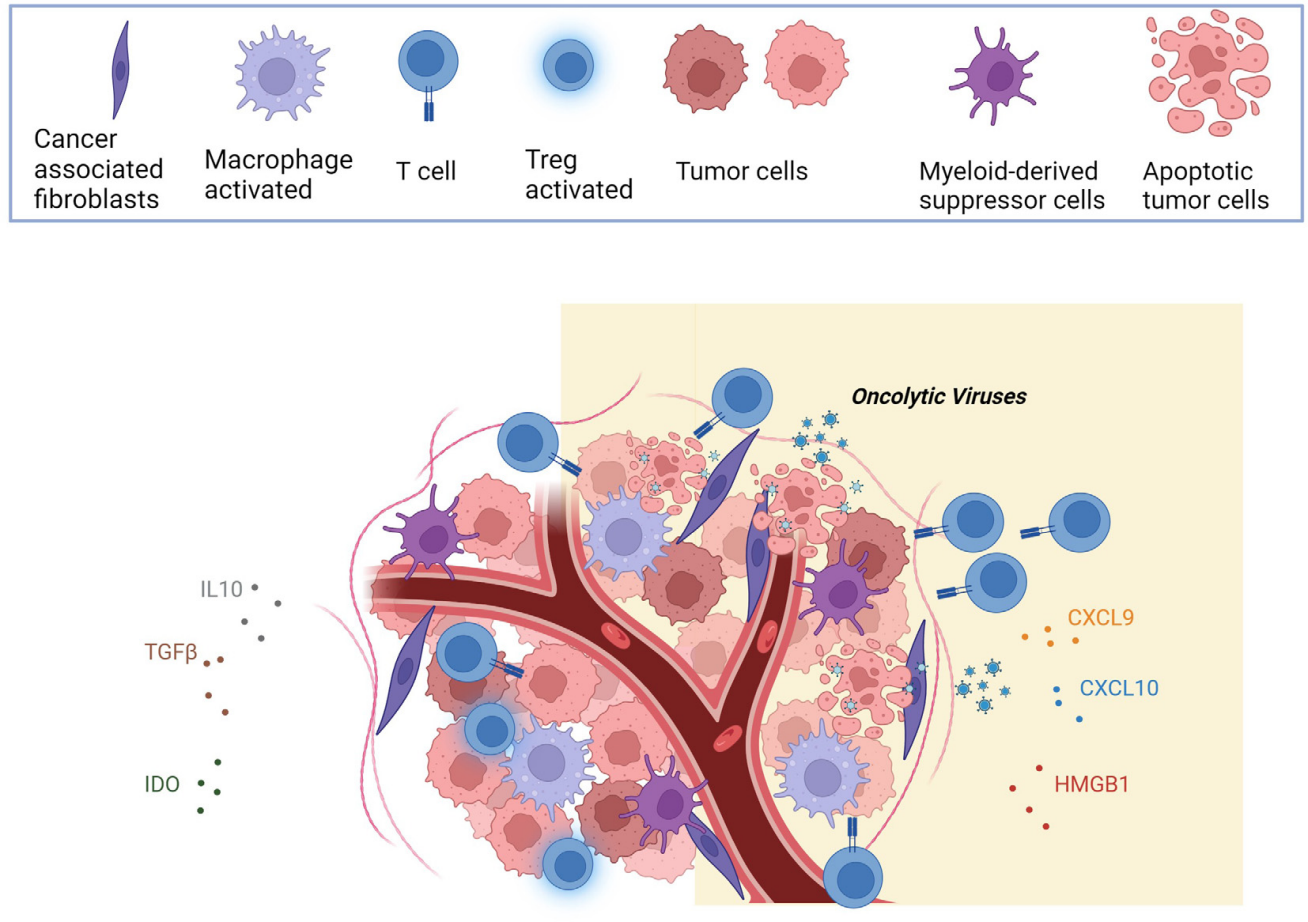
The figure illustrates that the heightened oncolytic immunogenicity is a distinctive feature of OVs. As OVs induce the lysis of tumor cells, they release a combination of viral progeny, tumor-specific antigens (TSAs), pathogen-associated molecular patterns (PAMPs), and damage-associated molecular patterns (DAMPs), orchestrating immunogenic cell death (ICD). This intricate process not only activates innate immunity, with collaboration between immune cells for effective tumor clearance. This leads to the release of inflammatory factors and chemokines (CXCL9, CXCL10, HMGB1), ultimately reversing the immunosuppressive characteristics of the tumor microenvironment (TME). Created with BioRender.com.

OVs induce oncolysis and immunogenic killing of tumor cells. OVs modulate specific cell death pathways such as apoptosis, autophagic cell death, necrosis, and necroptosis; many of these cell death pathways are by themself immunogenic (**Figure 1**). Moreover, OVs can be engineered to carry genes that produce immune-stimulating molecules, further boosting the anti-tumor response. These biological properties suggest that the combination of OVs with another immunotherapy agent may have considerable anti-tumor effects (42). Beyond OVs, CAR-T cells themselves can be engineered to express and deliver therapeutic factors. For instance, mesoCAR-T cells expressing anti-PD-1 antibodies are being evaluated in a phase II trial for treating mesothelin-positive advanced solid tumors (32). Additionally, IL-12-secreting Muc16-directed CAR-T cells have shown success in overcoming the immunosuppressive tumor microenvironment (TME) (43). Furthermore, OVs can be modified to express and deliver specific CAR antigens to the tumor surface, enhancing the targeting of corresponding CAR-T cells (44). In a study, a recombinant oncolytic vaccinia virus (OVV) was engineered to produce hyaluronidase (Hyal1) to break down hyaluronic acid in the tumor microenvironment. This degradation enhances drug delivery and immune cell infiltration, significantly improving antitumor effects both alone and in combination with other treatments (45). Recent research has found that combining oncolytic viruses with CAR-T cells enhances anti-tumor effects. However, delivering treatment to distant or metastatic tumors is challenging. To address this, scientists used CAR-T cells infected with HSV-1, which can systemically deliver the virus to solid tumors. These HSV-carrying CAR-T cells remained functional and, in mouse models, successfully delivered the virus to tumors, improving T-cell infiltration and significantly prolonging survival. The study demonstrates the potential of using CAR-T cells to target and treat distant tumors effectively (46). To improve CAR-T therapy for solid tumors, researchers used the A56 antigen, which is upregulated on tumor cells after administering an oncolytic vaccinia virus (OVV). A56-targeted CAR-T cells showed enhanced effectiveness in killing cancer cells *in vitro*. In mouse models, combining A56 CAR-T cells with OVV and hydroxyurea significantly reduced tumor size and delayed progression. This approach minimizes CAR-T effects on normal cells while improving targeting and treatment of various solid tumors. (47). Combining OVs with CAR-T cell therapy strategies could potentially function in a synergistic manner, offering complementary and additive benefits, that will be discussed in this review.

## 3 Impact of Ovs on the tumor microenvironment

The use of OVs, has emerged as a potentially powerful approach for cancer treatment by harnessing the immune system’s ability to recognize and eliminate tumor cells (48), OVs exploit several molecular and cellular mechanisms to enhance antitumor immunity. Firstly, OVs can selectively infect and replicate within cancer cells due to their inherent tumor cell tropism, which is achieved through specific receptor interactions or alterations in the tumor microenvironment (49). Upon viral replication, cancer cells undergo cytolysis, resulting in the release of tumor-associated antigens (TAAs) and danger signals (50). These TAAs are presented by antigen-presenting cells, such as dendritic cells (DCs), to activate tumor-specific T cells and initiate an adaptive immune response against the tumor. In addition to direct tumor cell killing, OVs induce immunogenic cell death (ICD), a process characterized by the release of immunostimulatory molecules. During ICD, cancer cells release damage-associated molecular patterns (DAMPs) such as calreticulin, high-mobility group box 1 (HMGB1), and adenosine triphosphate (ATP) (51). Cellular damage or stress is induced by various triggers, such as tissue injury, radiation, drug treatment, infection, and ischemic shock, leading to the release of DAMPs (52, 53). These DAMPs serve as danger signals that alert the immune system to the presence of dying tumor cells. They activate innate immune cells, including DCs, macrophages, and natural killer (NK) cells, which process TAAs, leading to antigen presentation and activation of cytotoxic T lymphocytes (CTLs) and other effector cells (54). This immune activation promotes tumor-specific immune responses and long-term memory against the tumor. Furthermore, OVs can be genetically engineered to express therapeutic transgenes that enhance the antitumor immune response. These transgenes can encode cytokines (e.g., interferons, interleukins) (55, 56), chemokines (e.g., CXCL9, CXCL10), immune checkpoint inhibitors (e.g., anti-PD-1 antibodies (56), or co-stimulatory molecules (e.g., CD40 ligand, 4-1BB ligand) (57). By expressing these immune-stimulatory molecules, OVs can augment immune cell recruitment, activation, and effector functions within the tumor microenvironment. For example, the expression of cytokines can promote the infiltration of immune cells into the tumor, enhance antigen presentation, and activate cytotoxic immune responses. Immune checkpoint inhibitors expressed by OVs can block inhibitory signalling pathways, such as PD-1/PD-L1, leading to the reinvigoration of exhausted T cells and restoration of antitumor immune responses (56). The combination of direct oncolysis, induction of ICD, and expression of immune-stimulatory molecules by OVs creates a highly immunogenic tumor microenvironment, facilitating the generation of systemic antitumor immunity. This multimodal approach not only targets and eliminates primary tumors but also promotes immune memory and helps to control metastatic disease. Moreover, as described below, OVs can be used in combination with other immunotherapies, such as immune checkpoint inhibitors or adoptive cell therapies, to achieve synergistic effects and enhance treatment outcomes.

By harnessing these mechanisms, oncolytic viruses (OVs) show immense potential for enhancing cancer treatment and broadening the therapeutic arsenal available for patients, including CAR-T cell therapy. Numerous preclinical studies have been published demonstrating the efficacy of this approach. (**Table 1**).

**Table 1:** Preclinical studies on oncolytic viruses and CAR-T cells.

Oncolytic Virus	Armed	References
Adenovirus	Onc.-Ad RANTES IL2	(58)
Adenovirus	CAdvec-αPDL1	(59)
Adenovirus	CAdvec-IL12 αPDL1	(60)
Adenovirus	BiTE CAdDuo interleukin [IL]-12 and PD-L1Ab	(61)
Adenovirus	Ad5/3-E2F-D24-TNFa-IRES-IL2, or OAd-TNFa-IL2	(62)
Adenovirus	oAD-IL7, B7H3-CAR-T	(63)
Adenovirus	CAd12_PDL1	(60, 64)
Vaccinia virus	VSVmIFNβ EGFRvIII CAR T	(65)
Vaccinia virus	CAR/CXCL11 VV.CXCL11	(66)
Herpes virus	oHSV T7011 with CD19 or BCMA CAR T-cell	(67)
Herpes virus	oHSV-1 CD70 CAR T	(68)

## 4 Enhancement of CAR-T cell function by OVs

**Figure 2:**
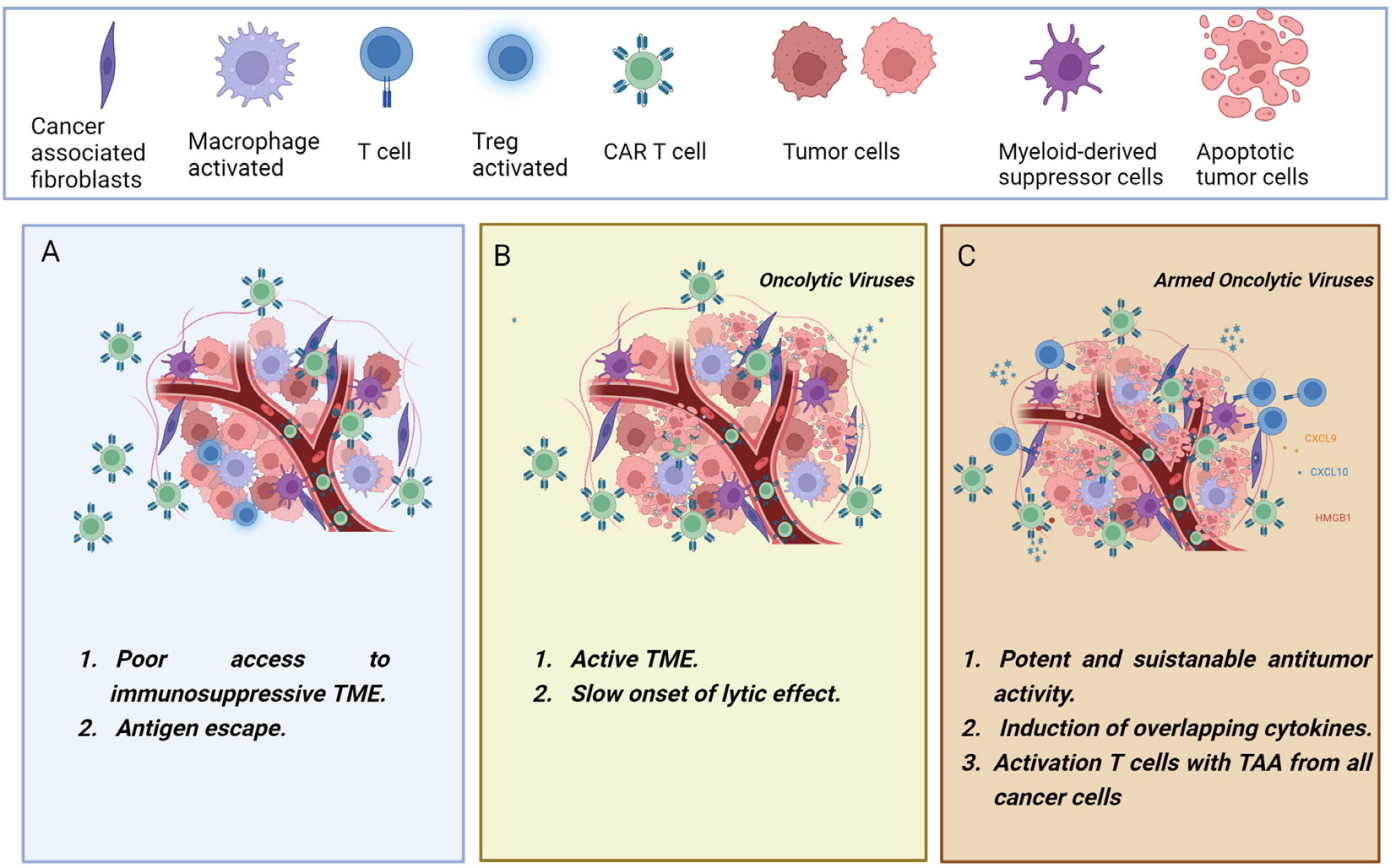
The figure illustrates the synergistic combination of CAR-T cells and oncolytic viruses. **(A)** CAR-T cells encounter various challenges in solid tumors, including an immunosuppressive environment that may lead to T cell dysfunction and treatment failure. **(B)** Administering oncolytic viruses for cancer treatment before CAR-T cell therapy leads to tumor debulking, immunogenic cell death, and a reversal of tumor immunosuppression. **(C)** In a collaborative effort, the engineered oncolytic viruses may transform the immunologically “cold” tumor into a “hot” tumor, exerting an upgraded and more powerful antitumor immunity. Oncolytic viruses can be genetically modified to deliver therapeutic transgenes into the tumor microenvironment, boosting T-cell effector functions. Combining CAR-T cells with oncolytic viruses armed with cytokines, chemokines, BiTEs, or immune checkpoint inhibitors has demonstrated enhanced therapeutic outcomes. Created with BioRender.com.

Setting up treatment strategies inducing complete therapeutic responses in patients with solid tumors presents significant challenges. However, OVs hold great promise as tools to overcome some of these obstacles and enhance the effectiveness of CAR-T cell therapy in solid tumors (**Figure 2**).

First, OVs can be armed with therapeutic transgenes to boost the CAR-T activation. Second, OVs could be able to survive and maintain their cytotoxicity functions in a tumor microenvironment that is immunosuppressed. This may provide danger signals that can revert tumor immunosuppression. Third, the direct lytic effect of OV on cancer cells results in tumor cell death and thus the release of tumor-associated antigens (TAA) (69).

Wing et al. described that the combination of an adenovirus vector (OAd) and BiTE treatment-mediated oncolysis, improved CAR-T cell activation and proliferation. Furthermore, this co-treatment increased cytokine production and cytotoxicity and showed a favourable safety profile *in vitro* compared to the EGFR- targeting CAR-T cells alone (70). Another opportunity is the combination of CAR-T cells with an armed oncolytic virus that delivers chemokine such as RANTES and IL15 to enhance the trafficking and persistence of the CAR-T cells, resulting in antitumor effects (71). It was described that infecting tumor cells with an oncolytic vaccinia virus coding for CD19t produced *de novo* CD19 at the cell surface before virus-mediated tumor lysis. Co-culture with CD19 CAR-T cells with OV19t produced secretion of cytokines and exhibited potent cytolytic activity against infected tumors (72). In another study, the authors used a combination of DD7-IL7 and B7H3 CAR-T *in vitro* and showed increased proliferation and persistence of tumor-infiltrating B7H3 CAR-T for glioblastoma treatment (63). The oncolytic adenovirus LOAd703, encoding CD40L and 4-1BBL transgenes, demonstrates promise in B cell tumor therapy, activating both antigen-presenting cells and T cells through CD46 interaction (73). When combined with CAR-T cell therapy, it elicits robust anti-lymphoma immune responses, significantly enhancing the effectiveness of CAR-T treatment (73). Additionally, oncolytic vaccinia viruses engineered to produce CXCL11 or armed with IL21 have shown potential in bolstering adoptive T cell transfer and vaccine-based immunotherapy, offering promising avenues for treatment improvement (66).

Furthermore, OVs can be modified to express and deliver specific CAR antigens to the tumor surface, enhancing the targeting of corresponding CAR-T cells (44). Looking ahead, combining oncolytic virotherapy with established treatments and emerging strategies like immune checkpoint inhibitors and CAR-T therapy holds considerable potential for future cancer treatment paradigms for solid tumors (74).

## 5 Examples of oncolytic viruses combined with CAR-T cell

### 5.1 Adenovirus

Adenoviruses (Ads) are small, non-enveloped viruses with a double-stranded DNA genome. One advantage of adenoviral vectors is that their genome remains episomal, conferring a safer profile compared to integrating viral vectors. Adenovirus-based genetic vaccines have demonstrated the ability to drive robust and sustained T cell and B cell responses against the encoded transgenes. This may be attributed to the vector’s ability to persist in a transcriptionally active form at the site of inoculation and in lymphatic tissues (75) Adenovirus, holds significant potential in the realm of CAR-T cell therapy (CAR-T). Adenoviral vectors can be genetically modified to efficiently deliver CAR genes into T cells, enabling the production of CAR-T cells (63, 76, 77). The high infectivity and transduction efficiency of adenoviruses make them an appealing tool for CAR-T cell manufacturing (78). By employing adenoviral transduction, CAR expression can be effectively achieved on the surface of T cells, empowering them to target and eliminate cancer cells. Furthermore, adenoviral vectors can be engineered to enhance CAR-T cell persistence and augment their anti-tumor activity (79). However, it is essential to address challenges such as pre-existing immunity against adenoviruses in the human population and potential toxicity associated with viral vectors. Ongoing preclinical (**Table 1**) and clinical investigations aim to optimize adenoviral vectors for CAR-T cell generation, with the goal of improving the safety and efficacy of CAR-T cell therapy across diverse cancer types (70).

### 5.2 Vaccinia virus

Vaccinia virus (VV) is a large, enveloped, double-stranded DNA virus that belongs to the Poxviridae family. It has been extensively used as a smallpox vaccine and has a well-established safety profile in humans. By leveraging its inherent oncolytic properties, vaccinia virus can selectively infect and replicate within cancer cells, triggering their destruction and has shown promise in clinical trials for advanced solid cancers (80). When combined with CAR-T cells, vaccinia virus can enhance the anti-tumor immune response by promoting the release of tumor antigens and creating an inflammatory milieu that supports CAR-T cell activation and cytotoxicity (65). Ongoing preclinical investigations will be important to explore the synergistic potential of vaccinia virus and CAR-T cells aim to optimize this combination therapy for improved outcomes in cancer treatment (66).

### 5.3 Herpes virus

Oncolytic herpes simplex virus (oHSV) have proven to be very potent OVs as they can selectively lyse tumor cells without harming healthy cells, addressing a key challenge in CAR-T cell therapy for solid tumors. Through genetic manipulation of the viral genome, oHSVs can be armed with various payloads such as cytokines, chemokines, and immune checkpoint inhibitors to convert immunologically “cold” tumors into “hot” tumors. Talimogene laherparepvec (T-VEC), the first clinically approved OV, is a genetically modified oHSV that expresses granulocyte-macrophage colony-stimulating factor (GM-CSF) and is utilized for treating melanoma (81). Possible combination with CAR-T cells are discussed in different papers. For instance, the authors Zhu et al. demonstrated that oncolytic herpes simplex virus type 1 (oHSV-1) enhances the therapeutic effect of CD70-specific CAR-T cells by promoting intratumoral T cell infiltration and the release of interferon-gamma (IFN-γ). This finding provides support for the incorporation of CAR-T therapy into glioblastoma (GB) therapeutic strategies (68). Moreover, *in vitro* and *in vivo* studies have demonstrated synergistic anti-tumor responses through combined treatment with oHSV T7011 and CAR-TCD19 or CAR-TBCMA cells (67). The efficacy assessments further validate the significant synergistic anti-tumor effects achieved by combining T7011 with either CAR-TCD19 or CAR-TBCMA cells across various solid tumor models. These findings collectively suggest that the next-generation oHSV T7011 holds substantial promise as a combinatorial therapy alongside CD19 or BCMA-specific CAR-T cells, offering a potential for the treatment of solid tumors (67).

## 6 Conclusion

The compelling potential of Onco-Immunotherapy, driven by the intricate molecular and cellular mechanisms inherent in oncolytic virus strategies, as discussed in the preceding sections of this review, strongly suggests that the synergistic use of CAR-T cells and OVs could be pivotal in overcoming tumor resistance and significantly improving therapeutic outcomes. When used in tandem, OVs can serve as powerful adjuvants to CAR-T cell therapy (71). They can sensitize tumors to CAR-T cell recognition by upregulating the expression of tumor antigens and immune-stimulatory molecules. This synergistic approach can address the limitations of CAR-T cell therapy in solid tumors and potentiate a more robust and durable anti-tumor immune response (82). OVs are genetically modified viruses designed to selectively infect and replicate within cancer cells. They possess several characteristics that make them attractive candidates for combination therapy with CAR-T cells. By infecting tumor cells, OVs can induce immunogenic cell death, release tumor antigens, and create an inflammatory environment favourable for CAR-T cell activation (83). Additionally, some OVs can directly modulate the tumor microenvironment, reversing immunosuppression and promoting an anti-tumor immune response. The integration of OVs with CAR-T cells in solid tumor treatment represents a promising avenue for future research and clinical applications (44). Ongoing studies are focused on optimizing the delivery methods, improving the safety profiles, and developing novel oncolytic viruses that can selectively target tumor cells while sparing normal tissues. Furthermore, combining these therapies with immune checkpoint inhibitors or other immunomodulatory agents may further enhance their effectiveness against most types of solid cancer. The combination of CAR-T cells and oncolytic viruses offers a promising strategy to address this hurdle. By harnessing the complementary mechanisms of action of these two modalities, researchers are striving to unlock the full potential of immunotherapy in the treatment of cancer. With continued advancements and clinical investigations, this combined approach holds great promise for the future of cancer treatment.
